# Nurse-led interventions in systemic autoimmune rheumatic diseases: a systematic review

**DOI:** 10.1186/s12912-023-01393-8

**Published:** 2023-07-04

**Authors:** Robyn K. Wojeck, Kimberly Arcoleo, Elizabeth C. Hathaway, Tamara J. Somers

**Affiliations:** 1 College of Nursing, University of Rhode Island, 350 Eddy St, Providence, RI USA; 2 Department of Psychiatry and Behavioral Sciences, School of Medicine, Duke University, 2400 Pratt St, Durham, NC USA

**Keywords:** Nurse-led intervention, Systemic autoimmune rheumatic disease, Patient-reported outcomes, Randomized controlled trials, Systematic review

## Abstract

**Background:**

Nurses play an important role in the management of patients with systemic autoimmune rheumatic diseases. Little is known about the effectiveness of nurse-led interventions on patient-reported outcomes in this population. The aim of this systematic review was to examine the evidence of nurse-led interventions in systemic autoimmune rheumatic diseases.

**Methods:**

Using the Preferred Reporting Items for Systematic Reviews and Meta-Analysis guidelines, a comprehensive literature search was conducted in PubMed, Cumulative Index to Nursing and Allied Health Literature, PsycINFO, and Embase for studies published from database inception to September 2022. Studies were included if they were published in a peer-reviewed journal in English and evaluated the effectiveness of a nurse-led intervention using a randomized controlled trial design in adults with a systemic autoimmune rheumatic disease. Screening, full-text review, and quality appraisal were conducted by two independent reviewers.

**Results:**

A total of 162 articles were identified for possible inclusion, of which five studies were included. Four of five studies (80%) were conducted in systemic lupus erythematosus. There was significant variability in the types of nurse-led interventions; the majority included educational sessions and follow up counseling by a nurse (n = 4). The most common patient-reported outcomes were health-related quality of life (n = 3), fatigue (n = 3), mental health (including anxiety and depression) (n = 2), and self-efficacy (n = 2). The duration of the interventions varied from 12 weeks to 6 months. All studies included a nurse with specialized training and education and showed significant improvements in their primary outcomes. The majority of the studies (60%) were considered high methodological quality.

**Conclusion:**

This systematic review provides emerging evidence for the use of nurse-led interventions in systemic autoimmune rheumatic diseases. Our findings emphasize the important role of nurses in providing nonpharmacological strategies to help patients better manage their disease and improve health outcomes.

**Supplementary Information:**

The online version contains supplementary material available at 10.1186/s12912-023-01393-8.

## Introduction

Systemic autoimmune rheumatic diseases (SARD) are a group of chronic autoimmune disorders characterized by immune dysregulation and inflammation affecting multiple organs, leading to disability and premature death [1]. Systemic autoimmune rheumatic diseases are among the most severe diseases affecting the musculoskeletal system [2] and include conditions such as systemic lupus erythematosus (SLE), systemic sclerosis (SSc), Sjogren’s syndrome, inflammatory myositis (i.e., polymyositis, dermatomyositis), and systemic vasculitides (i.e., giant cell arteritis, granulomatosis with polyangiitis, Takayasu’s disease, polyarteritis nodosa) [1]. There is no cure for this group of rheumatic diseases, and treatment is geared toward halting disease progression, ameliorating symptoms, and improving quality of life.

Patients with systemic autoimmune rheumatic diseases face a unique set of challenges, such as an unpredictable disease course, heterogeneity in clinical presentation, limited effective treatments, and variability in symptoms experienced [3]. This often leads to significant psychological distress, reduced physical function, and decrements in quality of life [4–6]. The treatment and management of systemic autoimmune rheumatic diseases is complex and requires specialty care. Patients with systemic autoimmune rheumatic diseases have described several challenges in receiving optimal rheumatology care, such as limited disease-specific information, lack of social support and coping resources, difficulty in obtaining care from their rheumatologist during symptom flares, and inaccessibility to a member of the healthcare team available between clinic visits to guide them in managing their rheumatic disease [3]. Additionally, receiving appropriate care for the management of these rheumatic diseases is hindered by a declining pool of specialists [7].

Nurses are particularly well-suited to address these challenges due to their essential role in the ongoing management and support of these patients [8]. Recently, the European League Against Rheumatism (EULAR) published its first evidence-based recommendations on the role of the nurse in the management of similar rheumatic diseases (i.e., chronic inflammatory arthritis) [8]. These recommendations described the nurse’s contribution to the management and care of patients with rheumatic diseases, including the nurse’s positive impact on providing patient education, disease management, psychosocial support, self-management, and improvements in access, satisfaction, and efficiency of care [9]. Additionally, these recommendations supported the use of nurse-led interventions, citing the effectiveness of these interventions on a number of outcomes such as self-efficacy, symptom severity, coping, physical and mental functioning, quality of care, disease status, and patient safety [9]. However, these recommendations did not encompass all rheumatic diseases, including systemic autoimmune rheumatic diseases. To our knowledge, no studies have systematically evaluated the use of nurse-led interventions in systemic autoimmune rheumatic diseases, limiting their inclusion in evidence-based recommendations such as those presented by EULAR.

To capture the unique and valuable contribution of nursing in rheumatology, it is imperative that we systematically examine the evidence of nurse-led interventions specific to systemic autoimmune rheumatic diseases. A better understanding of the impact of nurse-led interventions on patient-reported outcomes is needed to inform treatment guidelines and clinical practice in this population.

## Aims

The aims of this systematic review were to:

1. Examine the effectiveness and attributes of nurse-led interventions on patient-reported outcomes in individuals with systemic autoimmune rheumatic diseases.
2. Assess the nurse’s training and expertise in the delivery of the nurse-led interventions for patients with systemic autoimmune rheumatic diseases.

## Methods

### Design

This systematic review was completed using the Preferred Reporting Items for Systematic Reviews and Meta-Analyses (PRISMA) guidelines [10]. This systematic review was developed and registered in the International Prospective Register of Systemic Reviews (PROSPERO; registration pending approval).

### Search strategy

A comprehensive search was conducted in PubMed, Cumulative Index to Nursing and Allied Health Literature (CINAHL), PsycINFO, and Embase for studies published from database inception to September 2022. The searches included a combination of the following index and keywords using Boolean operators and truncation (*): systemic autoimmune rheumatic disease, nurse-led, nurse, and multidisciplinary (see supplementary Table 1). Reference lists were hand searched for relevant studies.

### Eligibility criteria

The aim of this review was to examine whether nurse-led interventions were effective (i.e., produced outcomes that were similar to or better than usual care) in patients with systemic autoimmune rheumatic diseases. As such, we decided *a priori* to include randomized controlled trials (RCTs) because RCTs are considered the gold standard in evaluating the effectiveness of an intervention, resulting in findings closer to the true effects than those produced by other research methods [11, 12]. While we acknowledge that the term systemic autoimmune rheumatic disease may include rheumatoid arthritis in other contexts, the focus of our systematic review was on those less common. Therefore, the definition of a systemic autoimmune rheumatic disease used this review included the following conditions: systemic lupus erythematosus (SLE), systemic sclerosis (SSc), Sjogren’s syndrome, inflammatory myositis (i.e., polymyositis, dermatomyositis), and systemic vasculitides (i.e., giant cell arteritis, granulomatosis with polyangiitis, Takayasu’s disease, polyarteritis nodosa). For the purposes of this systematic review, a nurse-led intervention was defined as any non-pharmacological intervention delivered by a nurse. This definition was adapted from EULAR’s review and recommendations on the role of the nurse in the management of chronic inflammatory arthritis [8]. While the keyword ‘multidisciplinary’ was also used to capture nurse-led intervention studies, interventions where the effect of the nurse delivering the intervention could not be isolated from those of a multidisciplinary team on the primary outcome were excluded [9].

Eligible studies were those that: (1) included patients diagnosed with a systemic autoimmune rheumatic disease (i.e., systemic lupus erythematosus, systemic sclerosis, Sjogren’s syndrome, polymyositis, dermatomyositis, giant cell arteritis, granulomatosis with polyangiitis, Takayasu’s disease, or polyarteritis nodosa), (2) were conducted in adults ≥ 18 years old, (3) described a RCT (4) included a nurse as the primary interventionist, (5) were published in English, and (6) were published in peer-reviewed journals. Studies were excluded if they were: (1) qualitative studies or other quantitative study designs (i.e., nonrandomized controlled trials, quasi-experimental, pre- and post-test design, comparative, prospective and retrospective cohort studies, case control studies, cross-sectional studies), (2) conference abstracts, editorial letters, comments, unpublished manuscripts, case reports, or literature reviews, (3) studies evaluating the effectiveness of a pharmacological treatment, (4) studies conducted in patients with other rheumatic diseases or chronic conditions, (5) studies in which the effect of the nurse could not be isolated, (6) studies with a sample including a systemic autoimmune rheumatic disease and another chronic condition in which the intervention effect on the systemic autoimmune rheumatic disease could not be differentiated from that of the total sample, (7) studies conducted in children or adolescents, (8) published in languages other than English, and (9) not available in full text.

### Study selection

**Fig. 1:**
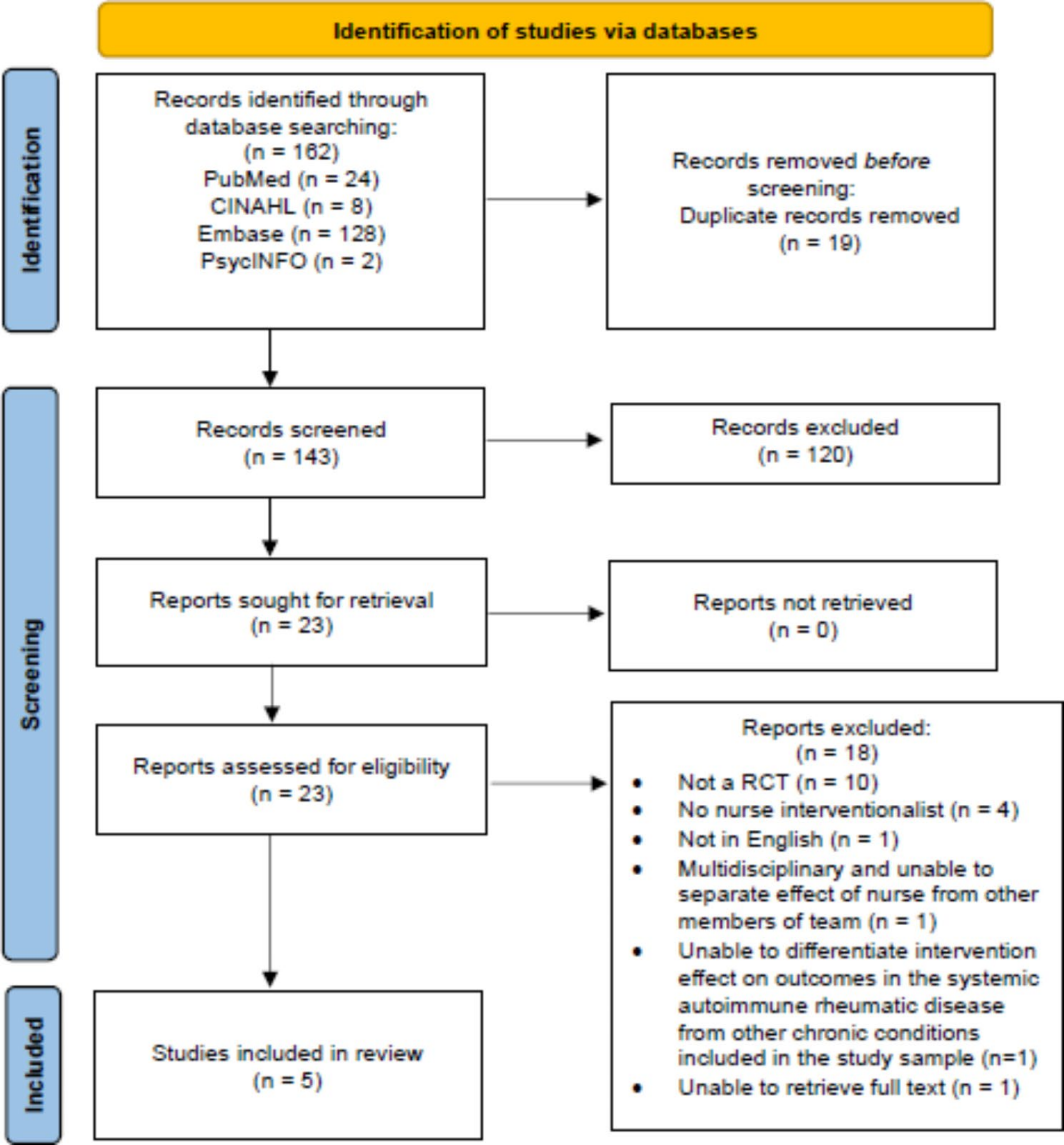
PRISMA Flow Diagram

A total of 162 articles were identified for possible inclusion. The retrieved articles were downloaded to a primary screening and data extraction tool (Covidence) and duplicates were removed. Titles and abstracts were screened by two reviewers (X.X., X.X.) to identify studies that met eligibility criteria. Full-text articles that met the inclusion criteria were reviewed by two authors (X.X., X.X.) and five articles were included in this review. Figure 1 provides the PRISMA flow diagram.

### Data extraction

Data extraction was guided by the JBI Reviewers Manual 2020 [13]. The following information was extracted and organized into tables: author, year, country, systemic autoimmune rheumatic disease studied, sample size and characteristics, overview of the intervention characteristics, comparator, setting, outcomes measured, and findings. Two reviewers (X.X., X.X.) independently assessed the extracted data and disagreements were resolved through discussion until consensus was achieved.

#### Quality assessment

The Joanna Briggs Institute (JBI) Critical Appraisal tool for randomized controlled trials [14] was used to evaluate the methodological quality of each study included in this systematic review. Responses to each of the thirteen items were marked “yes,” “no,” “unclear,” or “not applicable.” Study scores were determined by dividing the sum of the items marked “yes” by the total number of items in the JBI tool. The following study quality scores were used to determine study quality: low quality (< 60%), moderate quality (60–79%), and high quality (≥ 80%) [15–17]. Two reviewers (X.X., X.X.) independently evaluated the quality of the studies and disagreements were resolved through consensus.

## Results

### Study characteristics

Among the five studies, the majority (n = 4, 80%) were conducted in patients with systemic lupus erythematosus [18–21], followed by systemic sclerosis (n = 1) [22]. All study samples were predominately female [18–22]. Only one study reported race and ethnicity and the sample included mostly Caucasian females [20]. Studies were conducted in China, Italy, Taiwan, Turkey, and the United States of America (n = 1 each). Sample sizes in the intervention arm of the RCT ranged from 32 to 64. The majority of studies (n = 4, 80%) were conducted in the last five years [18, 19, 21, 22]. Table 1 provides an overview of the study characteristics.

**Table 1:** Overview of Study Characteristics

Author, year, country	SARD	Sample size	Sample characteristics				
			Age Mean (SD)	Female N (%)	Race/ethnicity N (%)	Marital status N (%)	Disease duration Mean (SD)
Kankaya & Karadakovan (2020); Turkey	SLE	Total = 80 Control = 40 Intervention = 40	Control: 39.0 (12.7) Intervention: 35.6 (8.4)	Control: 38 (95%) Intervention: 38 (95%)	N/S	N/S	Control:✝ < 1 yrs: 2 (5%) 1–10 yrs: 21 (52.5%) 11–20 yrs: 13 (32.5%) > 20 yrs: 4 (10%) Intervention:✝ < 1 yrs: 3 (7.5%) 1–10 yrs: 23 (57.5%) 11–20 yrs: 11 (27.5%) > 20 yrs: 3 (7.5%)
Karlson et al. (2004); US	SLE	Total = 122 Control = 58 Intervention = 64	Control: 40.8 (11.1) Intervention: 42.7 (22.8)	Control: 56 (98%) Intervention: 63 (98%)	White: Control: 48 (84%) Intervention: 57 (89%)	N/S	N/S
Uras et al. (2019); Italy	Systemic sclerosis	Total = 63 Control = 31 Intervention = 32	Control: 55.2 (13.3) Intervention: 54.6 (15.8)	100%	N/S	Control: 18 (58.1%) Intervention: 21 (65.6%)	Control: 11.8 yrs (8.4) Intervention: 8.7 yrs (6.3)
Wu et al. (2018); Taiwan	SLE	Total = 76 Control = 38 Intervention = 38	Control: 43.5 (12.7) Intervention: 43.8 (9.9)	100%	N/S	Control: 22 (57.9%) Intervention: 29 (76.3%)	Control: 12.3 yrs (8.4) Intervention: 12.0 yrs (7.4)
Xie et al. (2018); China	SLE	Total = 125 Control = 61 Intervention = 64	Control: 38.4 (15.8) Intervention: 35.9 (12.3)	Control: 54 (88.5%) Intervention: 57 (89.1%)	N/S	Control: 47 (77%) Intervention: 45 (70.3%)	Control: ≤ 3 yrs = 30 (49.2) > 3 yrs = 31 (50.8) Intervention: ≤ 3 yrs = 33 (51.6) > 3 yrs = 31 (48.4)

*Note.* SARD = systemic autoimmune rheumatic disease; SLE = systemic lupus erythematosus; N/S = not stated; yrs. = years; ✝ = data are shown as N (%) instead of mean (SD)

### Types of nurse-led interventions

There was significant variability in the types of nurse-led interventions reported among the five studies (Table 2). The most common intervention included educational sessions and follow up counseling by a nurse (n = 4) [18, 20–22]. The remaining study evaluated a transitional care program after hospital discharge [19]. The duration of the nurse-led interventions varied from 12 weeks to 6 months, with the majority of interventions (n = 3, 60%) taking place for 6 months or greater [18, 20, 22]. Almost all interventions included a face-to-face component (n = 4, 80%) [19–22] and were primarily delivered in an outpatient setting (i.e., outpatient clinic or at home) (n = 4, 80%) [18–21].

**Table 2:** Intervention Characteristics and Study Findings

Author	Nurse’s training and expertise	Frequency of contact with the nurse (#/times)	Intervention overview	Intervention description	Comparator	Duration of intervention	Follow up time frame	Primary setting (inpatient vs. outpatient)	Outcomes & Measures	Findings
Kankaya & Karadakovan	Nurse researcher who specialized in the field	Dependent on the counseling needed as determined by the participant	Web-based education followed by individual nurse consultancy	3 months of web-based education on self-management of SLE, followed by 6 months of individual nurse consultancy.	Usual care	6 mo.	6 mo.	Outpatient	Self-efficacy (SEMCD) Fatigue (FSS) Assessment/Satisfaction of care (PACIC) Disease activity (SLEDAI-2000)	Significant improvements in self-efficacy, fatigue, and assessment of chronic illness care in the intervention group
Karlson et al.	Masters-level nurse with 20 years of clinical experience, was trained in proven efficacy-enhancing counseling techniques	N = 7 (1 in person, 6 counseling telephone sessions)	Educational session followed by counseling telephone calls	Intervention designed to enhance self-efficacy, couple’s communication about lupus, social support, and problem solving, in the form of a 1-hour session with a nurse educator followed by monthly telephone counseling for 6 months with the dyads.	Attention placebo (45 min. video and monthly telephone calls)	6 mo.	12 mo.	Outpatient	General health status (SF-36) •Global physical function •Global mental health status SLE disease activity (SLAQ) Fatigue (SF-36 vitality subscale, SLAQ) Self-efficacy (self-efficacy-other scale) Social support (modified social support scale) Problem solving (11-item coping measure) Satisfaction with medical care (MISS) Compliance (4-item adherence measure)	Significant improvements in couples’ communication, problem-focused coping, social support, self-efficacy, fatigue, and global mental and physical health status
Uras et al.	Research nurse who was an expert in adult education	N = 4 (during hospital admission, 3, 6, 9 and 12 months)	Daily oral exercise with nurse support	Educational materials provided initially followed by face-to-face interventions with a nurse to teach oral exercises which patients were expected to perform daily and record on a diary card.	Usual care	12 mo.	3, 6, 9, & 12 mo.	Inpatient	Mouth opening (measured in centimeters using calipers) Mouth disability (MHISS) Health status (SYSQ) QOL (Skindex-17) Anxiety & depression (GHQ-12)	Statistically significant increase in mouth opening compared to control group (using per-protocol analysis)
Wu et al.	Doctorally prepared nurse (PhD) with expertise in physical activity counselling for patients with SLE, and had been trained in counselling techniques	N = 6 (3 in-person, 3 phone calls)	Educational sessions with counseling telephone calls	Exercise counselling program and wore a pedometer on the waist for 1 week as the baseline and for 12 weeks following the baseline. Received face-to-face counseling at weeks 1, 4, and 8, as well as follow up phone calls to address barriers and see how daily goals were being met.	Given pedometer and instructions to wear for 12 weeks after 1 week baseline. Received usual care and phone calls with medication or lab information only.	13 wks.	8 & 12 wks.	Outpatient	Physical activity (Agoss Health Pedometer) Disease activity (SLEDAI-2000) HRQOL (SF-36) Fatigue (FSS) Sleep quality (PSQI)	Significant improvements in daily steps, quality of sleep, vitality, and mental health
Xie et al.	2 nurses with master’s degree and experience with SLE. Both trained in transitional care and Omaha system knowledge/skills	N = 8 (4 structured assessments and 4 telephone follow-ups)	Transitional care program following admission to hospital	In-person contact 1, 4, 8, and 12 weeks after hospital discharge to identify problems and introduce interventions. Telephone follow-up at 2, 3, 6, and 10 weeks after discharge to counsel patients regarding concerns about disease barriers.	Usual care	12 wks.	90 days from discharge	Outpatient once discharged from the hospital	Disease activity (SLEDAI-2000) Self-care (ESCA) QOL (SF-36) 30-day hospital readmission rate	Significant improvements in self-care and QOL. The 30-day readmission rate for patients in the intervention group were significantly lower than usual care group.

*Note.* SLE = systemic lupus erythematosus; mo. =months, wks. =weeks; SEMCD = Self-Efficacy for Managing Chronic Disease; FSS = Fatigue Severity Scale; PACIC = Patient Assessment of Chronic Illness Care; SLEDAI-2000 = SLE Disease Activity Index; SF-36 = Medical Outcomes Study 36-Item Short Form Survey; SLAQ = Systemic Lupus Activity Questionnaire; MISS = Medical Interview Satisfaction Scale; MHISS = Mouth Handicap in Systemic Sclerosis; SYSQ = Systemic Sclerosis Questionnaire; GHQ-12 = General Health Questionnaire; PSQI = Pittsburgh Sleep Quality Index; ESCA = Exercise of Self-Care Agency Scale

### Nurses delivering the intervention

All studies included a nurse with specialized training and education (Table 2). Two studies included a Master’s prepared nurse [19, 20], and one study utilized a doctorally prepared nurse as the interventionalist [21]. The majority of studies described the nurse’s previous training relevant to the delivery of the intervention (e.g., training in transitional care, counseling techniques) (n = 3, 60%) [19–21]. The frequency of contact with the nurse interventionist was reported in four out of the five studies and ranged from four to eight point of contacts [19–22].

### Nurse-led intervention outcomes and their effectiveness

A variety of outcomes evaluated the effectiveness of the nurse-led interventions (Table 2). The most common patient-reported outcomes were health-related quality of life (n = 3) [19, 21, 22], fatigue (n = 3) [18, 20, 21], mental health (including anxiety and depression) (n = 2) [20, 22], and self-efficacy (n = 2) [18, 20]. An array of measurement tools were used, of which the 36-Item Short Form Survey (SF-36) was the most common (n = 3) [19–21]. All studies showed significant improvement in their primary outcomes (see Table 2). Among the patient-reported outcomes included in the five studies, nurse-led interventions most frequently improved fatigue/sleep quality (n = 3) [18, 20, 21] and mental health (n = 3) [19–21].

### Quality appraisal of the studies

Table 3 provides an overview of the quality appraisal for all studies. The majority of the studies (n = 3, 60%) were considered high methodological quality [19, 21, 22]. The remaining studies were considered moderate quality [18, 20]. Among the studies with moderate methodological quality [18, 20], criteria related to the blinding of those delivering the intervention as well as outcome accessors was not stated. No studies were considered low methodological quality.

**Table 3:** Quality Appraisal of the Included Studies

Citation	Q1	Q2	Q3	Q4	Q5	Q6	Q7	Q8	Q9	Q10	Q11	Q12	Q13	Score	Quality Level
Kankaya & Karadakovan	Y	Y	Y	N	U	U	Y	Y	Y	Y	Y	Y	Y	10/13 (76.9%)	Moderate
Karlson et al.	Y	Y	Y	U	U	U	Y	Y	Y	Y	Y	Y	Y	10/13 (76.9%)	Moderate
Uras et al.	Y	Y	Y	Y	N	Y	Y	Y	Y	Y	Y	Y	Y	12/13 (92.3%)	High
Wu et al.	Y	Y	Y	Y	N	Y	Y	Y	Y	Y	Y	Y	Y	12/13 (92.3%)	High
Xie et al.	Y	Y	Y	N	N	Y	Y	Y	Y	Y	Y	Y	Y	11/13 (84.6%)	High

*Note.* The following study quality scores were used to determine study quality: low quality (< 60%), moderate quality (60–79%), and high quality (≥ 80%)

## Discussion

To our knowledge, this systematic review was the first to examine the effectiveness of nurse-led interventions in systemic autoimmune rheumatic diseases. All studies demonstrated significant improvements in their primary outcome, of which improvements in fatigue/sleep quality and mental health were the most common. At a time when there is a shortage of rheumatology specialists, nurses remain an untapped resource in the delivery of nonpharmacological interventions such as those included in this review. Previous research has described the breadth of rheumatology nursing activities, from disease assessment, patient education, support, symptom management, and prevention of disease complications [9]. This systematic review captures this broad range of nursing activities as demonstrated by the delivery of a multitude of nurse-led interventions that included educational sessions, counseling, exercise programs, and transitional care programs.

Our findings are particularly important when, within rheumatology, nurse-led care continues to grow as a model of care delivery due to a global shortage of rheumatologists and an increased need for patient education, monitoring, and support [7]. Nurse-led care is defined as care delivered by nurses with advanced competence in their specialty and who function either independently and/or interdependently with a multidisciplinary healthcare team [23]. Interventions commonly employed under this care model include those that focus on the assessment and evaluation of a person’s health condition and its symptoms, as well as those that include health teaching or counseling to manage symptoms and prevent complications [23]. This care delivery model has improved patient-centered care [24], as well as quality of life, fatigue, and patient knowledge [25] in patients with other rheumatic conditions. Additionally, nurse-led care has been found to be comparable or even more cost-effective than other models of care in rheumatic diseases [26]. In this systematic review, the interventions delivered by the nurses were similar to those frequently performed in nurse-led models of care, and all studies demonstrated positive effects on their primary outcomes, providing critical evidence supporting nurse-led interventions in systemic autoimmune rheumatic diseases. Future research is needed to evaluate the cost-effectiveness of these interventions in less common systemic autoimmune rheumatic diseases, such as those included in this review.

Patients have reported the valuable contribution of nurses in managing their rheumatic disease [27]. Our findings support the positive contribution of nurses in delivering effective interventions that improve patient-reported outcomes. However, the nurse’s role in rheumatology remains ambiguous and varies across the world due to a lack of standardized educational requirements and competencies. In 2012, the European League Against Rheumatism sought to provide recommendations on the role of the nurse in the management of chronic inflammatory arthritis to improve and standardize professional nursing care across Europe [8]. A main limitation identified in these recommendations was the lack of high-quality studies with clear descriptions of nursing roles and interventions [8]. To our knowledge, this was the first systematic review to address this critical gap in knowledge by systematically evaluating the specific components of nurse-led interventions in systemic autoimmune rheumatic diseases, as well as the nurse’s specific training and expertise in delivering these types of interventions.

This systematic review had several strengths and limitations. Two reviewers used a systematic approach to screen all titles, abstracts, and full-text articles, as well as conduct the quality appraisal for all studies. This is especially important to minimize errors and improve the credibility of our findings. The studies included in this systematic review were considered moderate to high methodological quality, providing support for the inclusion of nurse-led interventions in evidence-based guidelines for systemic autoimmune rheumatic diseases. The main limitation impacting the overall methodological quality of these studies was related to the lack of blinding of the nurse to the patients’ treatment assignment, which is particularly challenging in nurse-led interventions due to the nurse’s primary role in delivering the intervention.

Another limitation is that qualitative studies were excluded, limiting our understanding of the overall effectiveness of nurse-led interventions from the patient perspective or other lenses. In addition, the inclusion of studies in this systematic review may have been limited by publication bias. Given the heterogeneity among the five studies, we were unable to perform a meta-analysis and quantitatively evaluate for possible publication bias among the included studies. Moreover, there was a paucity of RCTs (i.e., only five studies), highlighting the need for more high quality, rigorous study designs to evaluate the effectiveness of nurse-led interventions in systemic autoimmune rheumatic diseases. Only studies that were published in English were included, limiting the generalizability of our findings. Lastly, studies might not have been captured due to inconsistent database indexing and variability in keywords and search terms, potentially leading to missed studies.

## Conclusion

This systematic review evaluated the effectiveness of nurse-led interventions in systemic autoimmune rheumatic diseases and the attributes of the nurses delivering these interventions. This review provides emerging evidence for the use of nurse-led interventions in this population and underscores the important role nurses have in the management of patients with systemic autoimmune rheumatic diseases. Our findings provide a foundation for the development of recommendations on the role of the nurse in systemic autoimmune rheumatic diseases. Future research is needed to comprehensively and rigorously examine the impact of nurse-led interventions through conduct of RCTs, the inclusion of qualitative studies, and assessment of other health outcomes in this population.

## Electronic supplementary material

Below is the link to the electronic supplementary material.

Supplementary Material 1

## Data Availability

All data generated or analyzed during this study are included in this published article and its supplementary information files.
