# Supplementary material for: Nurse-led interventions in systemic autoimmune rheumatic diseases: a systematic review
Source: BMC Nurs. 2023 Jul 4;22:232. doi: 10.1186/s12912-023-01393-8 (PMC10318744; doi:10.1186/s12912-023-01393-8)
Supplement: Supplementary file 1 — Supplementary Material 1 [file 12912_2023_1393_MOESM1_ESM.docx]

**Supplementary Data.**

Table S1. Search strategy for each database

| PubMed | (("systemic lupus erythematosus" OR "systemic sclerosis" OR "systemic scleroderma" OR "polymyositis" OR "dermatomyositis" OR "Sjogren's syndrome" OR "giant cell arteritis" OR "churg-strauss syndrome" OR "polyarteritis nodosa" OR "granulomatosis with polyangiitis" OR "takayasu arteritis" OR "Lupus Erythematosus, Systemic"[Mesh] OR "Scleroderma, Systemic"[Mesh] OR "Polymyositis"[Mesh] OR "Dermatomyositis"[Mesh] OR "Sjogren's Syndrome"[Mesh] OR "Giant Cell Arteritis"[Mesh] OR "Churg-Strauss Syndrome"[Mesh] OR "Polyarteritis Nodosa"[Mesh] OR "Granulomatosis with Polyangiitis"[Mesh] OR "Takayasu Arteritis"[Mesh]) AND (("Nurses"[Mesh] OR "Patient Care Team"[Mesh] OR "nurs*" OR "multidisciplinary"))) AND (randomized controlled trial or clinical trial.pt) |
| --- | --- |
| CINAHL | (MH "Churg-Strauss Syndrome") OR (MH "Dermatomyositis") OR (MH "Giant Cell Arteritis") OR (MH "Polyarteritis Nodosa") OR (MH "Polymyositis") OR (MH "Scleroderma, Systemic") OR (MH "Sjogren's Syndrome") OR (MH "Takayasu Arteritis") OR (MH "Wegener's Granulomatosis") OR (MH "Lupus Erythematosus, Systemic")  OR "systemic lupus erythematosus" OR "systemic sclerosis" OR "systemic scleroderma" OR "polymyositis" OR "dermatomyositis" OR "Sjogren's syndrome" OR "giant cell arteritis" OR "churg-strauss syndrome" OR "polyarteritis nodosa" OR "granulomatosis with polyangiitis" OR "takayasu arteritis" AND ((MH "Nursing Interventions") OR (MH "Nursing Care") OR (MH "Patient Care") OR (MH "Multidisciplinary Care Team") OR “nurs*” OR “multidisciplinary”)) |
| Embase | ('systemic lupus erythematosus'/exp OR 'systemic lupus erythematosus' OR 'systemic sclerosis'/exp OR 'systemic sclerosis' OR 'systemic scleroderma'/exp OR 'systemic scleroderma' OR 'polymyositis'/exp OR 'polymyositis' OR 'dermatomyositis'/exp OR 'dermatomyositis' OR 'sjogren syndrome'/exp OR 'sjogren syndrome' OR 'giant cell arteritis'/exp OR 'giant cell arteritis' OR 'churg-strauss syndrome'/exp OR 'churg-strauss syndrome' OR 'polyarteritis nodosa'/exp OR 'polyarteritis nodosa' OR 'granulomatosis with polyangiitis'/exp OR 'granulomatosis with polyangiitis' OR 'takayasu arteritis'/exp OR 'takayasu arteritis') AND ("nurs*" OR "multidisciplinary”) |
| PsycINFO | (DE "Lupus" OR "systemic lupus erythematosus" OR "systemic sclerosis" OR "systemic scleroderma" OR "polymyositis" OR "dermatomyositis" OR "Sjogren's syndrome" OR "giant cell arteritis" OR "churg-strauss syndrome" OR "polyarteritis nodosa" OR "granulomatosis with polyangiitis" OR "takayasu arteritis") AND (DE "Nursing" OR DE "Nurses" OR DE "Interdisciplinary Treatment Approach" OR DE "Interdisciplinary Research" OR "nurs*" OR "multidisciplinary") |
